# Anemoside B4 Protects against Acute Lung Injury by Attenuating Inflammation through Blocking NLRP3 Inflammasome Activation and TLR4 Dimerization

**DOI:** 10.1155/2020/7502301

**Published:** 2020-12-03

**Authors:** Renyikun Yuan, Jia He, Liting Huang, Li-Jun Du, Hongwei Gao, Qiongming Xu, Shilin Yang

**Affiliations:** ^1^College of Pharmacy, Guangxi University of Chinese Medicine, Nanning 530000, China; ^2^State Key Laboratory of Innovative Drug and Efficient Energy-Saving Pharmaceutical Equipment, Jiangxi University of Traditional Chinese Medicine, Nanchang 330004, China; ^3^Guangxi Engineering Technology Research Center of Advantage Chinese Patent Drug and Ethnic Drug Development, Nanning 530020, China; ^4^School of Life Sciences, Tsinghua University, Beijing 100084, China; ^5^College of Pharmaceutical Science, Soochow University, Suzhou 215123, China

## Abstract

Acute lung injury (ALI) is an acute inflammatory process in the lung parenchyma. Anemoside B4 (B4) was isolated from Pulsatilla, a plant-based drug against inflammation and commonly applied in traditional Chinese medicine. However, the anti-inflammatory effect and the mechanisms of B4 are not clear. In this study, we explored the potential mechanisms and anti-inflammatory activity of B4 both *in vitro* and *in vivo*. The results indicated that B4 suppressed the expression of iNOS, COX-2, NLRP3, caspase-1, and IL-1*β*. The ELISA assay results showed that B4 significantly restrained the release of inflammatory cytokines like TNF-*α*, IL-6, and IL-1*β* in macrophage cells. In addition, B4 rescued mitochondrial membrane potential (MMP) loss in (lipopolysaccharide) LPS plus ATP stimulated macrophage cells. Co-IP and molecular docking results illustrated that B4 disrupted the dimerization of TLR4. For *in vivo* results, B4 exhibited a protective effect on LPS and bleomycin- (BLM-) induced ALI in mice through suppressing the lesions of lung tissues, the release of inflammatory cytokines, and the levels of white blood cells, neutrophils, and lymphoid cells in the blood. Collectively, B4 has a protective effect on ALI *via* blocking TLR4 dimerization and NLRP3 inflammasome activation, suggesting that B4 is a potential agent for the treatment of ALI.

## 1. Introduction

Acute lung injury (ALI), a disease that abruptly or rapidly loses lung function, is caused by an aberrant inflammatory response and leads to death [1]. ALI always leads to the proliferation of pulmonary interstitial, alveolar edema, and acute hypoxia, which is involved in an acute inflammatory process in the lung. The pathophysiological characteristics are acute hypoxemia, respiratory distress, and pulmonary exudative lesions in the clinic, which progresses to acute respiratory distress syndrome (ARDS) [2]. Previous studies indicated that the proinflammatory cytokines such as TNF-*α*, IL-6, and IL-1*β* are involved in regulating and promoting the lung injury process. The release of cytokines will contribute to activating the acute lung injury inflammation process [3]. Therefore, exploring the inflammatory process and mechanisms of ALI are essential for understanding this terrible clinical syndrome [4], which is beneficial to search for a potential agent for protecting ALI/ARDS.

The inflammasome is a macromolecular complex that is activated by caspase-1 and facilitates the maturation of IL-1*β* and IL-18 [5]. NLRP3 inflammasome is a member of the NLR family, which is compose of NLRP3, ASC, and procaspase-1 [6]. There are two steps to activate the NLRP3 inflammasome. The first step is to prime the macrophages with pathogen-associated molecular patterns (PAMPs), which is recruited to the specific pattern recognition receptors (PRRs) [7], like LPS, a well-known PAMP-expressed Gram-negative bacteria. LPS binds to TLRs, leading to upregulation of the inflammasome components [8] to promote the pro-IL-*β* and NLRP3 transcription [9]. The second step is to form the NLRP3 inflammasome. The NLRP3 inflammasome can be activated by numerous stimuli, including ATP, nigericin [10], alum, bacterial, and viral pathogens [11–13]. Following the assembly of inflammasomes, the intracellular caspase-1 is activated, promoting the maturation of IL-*β* [14]. Previous studies showed that the activation of NLRP3 inflammasome induced by ATP leads to the loss of mitochondrial membrane potential, the activation of caspase-1, and the release of IL-1*β* [15], which are involved in the ALI inflammatory [16, 17].

Macrophages are critical mediators of innate immune responses [18]. In the process of inflammation, macrophages participate in regulating the immune and inflammatory response, which plays an important role in the development and recovery of ALI [19]. Macrophages always express TLR4 on the cell surface, the receptor of LPS, which regulates the innate immune response and the release of inflammatory cytokines [20]. When macrophage cells are activated by LPS, TLR4-MD-2 will form a dimerization and activate its downstream MyD88 to regulate nuclear factor-*κ*B (NF-*κ*B) or mitogen-activated protein kinase (MAPK) signaling pathways [21–23], leading to the release of proinflammatory factors [24, 25]. It has been reported that TLR4 is also associated with NLRP3 inflammasome when ALI happens [26]. Therefore, exploring a potential agent for the treatment of ALI, of which the mechanism is involved in the TLR4 and NLRP3 inflammasome, is an alternative strategy.

*Pulsatilla chinensis* (Bge.) Reg (Pulsatilla) is a traditional Chinese medicine, of which clinical indications mainly focus on fever and dysentery, in the light of its clearing heat, detoxicating, and blood cooling activities. Previous researches indicated that *P. chinensis* exerts antimicrobial, antitumor, and anti-inflammatory effects [27–30]. Recent studies found that *P. chinensis* has a therapeutic effect on lung cancers [31, 32], indicating that *P. chinensis* could have value as an agent of treating lung diseases.

Anemoside B4 (B4), a triterpenoid saponin, is quantized over 4.6% in the raw roots of *Bai-Tou-Weng* in terms of the 2015 Edition of Chinese Pharmacopoeia [33]. Although previous studies showed that B4 exhibits antimicrobial, antiviral, antitumor, and anti-inflammatory effects [34–37], quite few studies involving B4's protective effects on ALI are found. Therefore, in this study, we used B4 to explore its effect on ALI and firstly identified the protective effects on ALI and the underlying mechanisms of B4 both *in vitro* and *in vivo*.

## 2. Materials and Methods

### 2.1. Cell Culture

THP-1 cells are a gift from Dr. Lijuan Liu (Institute of Chinese Medical Sciences, University of Macau), which are bought from the American Type Culture Collection (ATCC, Manassas, VA, USA); J774A.1 cells were obtained from the Kunming Cell Bank of the Chinese Academy of Sciences (Kunming, China). THP-1 and HEK293T were bought from the American Type Culture Collection (ATCC, Manassas, VA, USA). J774A.1 cells and HEK293T cells were cultured in DMEM with 10% FBS. THP-1 cells were cultured in RPIM 1640 with 10% FBS. Cells were incubated in a 37°C, 5% CO_2_ incubator.

### 2.2. MTT Assay

J774A.1 and THP-1 cells were plated in 96-well plates with a density of 5 × 10^4^ for 24 h, then treated with B4 for another 24 h. The cell viability was determined by MTT.

### 2.3. Assessment of Inflammatory Cytokines

IL-1*β*, TNF-*α*, and IL-6 were detected by ELISA assay. THP-1 cells were plated into 24-well plates (2 × 10^5^, 24 h). Pretreatment with B4 (5, 10, and 20 *μ*M) was for 4 h, cocultured with LPS (1 *μ*g/mL) for another 5 h, then stimulated with ATP (5 mM) for 30 min. The supernatant was collected and employed for the detection of TNF-*α*, IL-6, and IL-1*β* release.

### 2.4. Detection of Mitochondrial Membrane Potential (MMP)

J774A.1 and THP-1 cells (5 × 10^4^ cells/well) were plated in 96-well culture plates and cultured in an incubator overnight. Pretreatment with B4 (20 *μ*M) for 4 h, then the cells cultured with 1 *μ*g/mL LPS for an additional 5 h and stimulated with ATP (5 mM) for 30 min. JC-1 staining (10 *μ*g/mL) was done for 30 min in an incubator in the dark. The images were taken by fluorescence microscopy (Leica, Wetzlar, Germany).

### 2.5. Western Blotting Assays

J774A.1 and THP-1 cells' lyses were collected; SDS-page and immunoblotting were described in our previous study [25]. The first antibodies and second antibodies were diluted into 1 : 1000 and 1 : 10000, respectively. The signals of chemiluminescence intensity were determined with SuperSignal™ West Femto Maximum Sensitivity Substrate and ChemiDoc™ MP Imaging System.

### 2.6. The Formation of TLR4 Dimer

HEK293T cells were plated in the dish (4 × 105, 10 cm i.d.) overnight. The plasmids were purchased from Addgene (Beijing, China) and cotransfected into HEK293T cells for 24 h. The transfected cells were treated with B4 (20 *μ*M) for 1 h, then cocultured with LPS (1 *μ*g/mL) for 12 h. Cells were harvested, and Western blotting was used to analyze the formation of TLR4 dimerization.

### 2.7. Docking of TLR4 Dimerization

The molecular docking data of B4 with the TLR4-MD-2-LPS complex (PDB code: 3FXI) was performed in LeDock (http://www.lephar.com). TLR4-MD-2-LPS complex structures were obtained from the RCSB Protein Data Bank (PDB code: 3FXI) [38].

### 2.8. Animal Experiments

In this study, the animal experiments were approved by the Ethics Committee of the Experimental Animal Centre of Guangxi University of Chinese Medicine (Nanning, China, No. SYXK-GUI-2019-0001). All the experiments in this study were in agreement with the Local Guide for the Care and Use of Laboratory Animals. The male BALB/c mice (6-8 weeks, 20 ± 2 g) were purchased from the Beijing Vital River Laboratory Animal Technology Co., Ltd. (Beijing, China). All mice were housed under standard (SPF) conditions with air filtration.

In the LPS-induced sepsis mouse model experiment, mice were divided into a control group, LPS model group (15 mg/kg, i.t.), B4 groups (2.5, 5, and 10 mg/kg, i.v.), and dexamethasone (DEX, 5 mg/kg, i.p.) group. The experimental group mice were administrated with noninvasive intratracheal instillation of LPS into the lung tissue. The B4 group mice were injected with B4 at 0, 3, 24, 48, and 72 h after LPS administration. The DEX group mice were injected with DEX after LPS treatment. The survival rate of mice was observed for 144 h. After 144 h, the mice were sacrificed.

In the LPS-induced ALI model, the model group mice were administrated with noninvasive intratracheal instillation of LPS (4 mg/kg, i.t.) into the lung tissue. In B4-treated groups, mice were injected with B4 (2.5, 5, and 10 mg/kg, i.v.) at 0, 3, 24, 48, and 72 h after LPS administration. The DEX group mice were injected with DEX (5 mg/kg, i.p.) after LPS treatment. Blood samples were collected, and the levels of lymphocyte (LYMPH), neutrophil (NEUT), and white blood cell (WBC) were determined by an automatic blood cell analyzer for animals (Mindray, Shenzhen, China). Serum and lung tissues were collected for the determination of cytokines, like TNF-*α*, IL-6, IL-1*β*, and MPO. Furthermore, the right lung tissues were fixed in 10% formaldehyde. The tissues were stained with H&E and imaged by a microscope (Leica, Wetzlar, Germany).

In the BLM-induced ALI model, the model group mice were administrated by noninvasive intratracheal instillation of BLM (3 mg/kg, i.t.) into the lung tissue. The experiment group mice were treated with B4 (2.5, 5, and 10 mg/kg, i.v.) and pirfenidone (PFD, 300 mg/kg, i.g.). After BLM injection, the B4-treated group mice were injected with B4 at 0, 3, 24, and 48 h. The PFD group mice were administrated with PFD (300 mg/kg, i.g.) at 0, 3, 24, and 48 h after BLM treatment. Blood samples were collected, and the levels of NEUT were determined by an automatic blood cell analyzer for animals (Mindray, Shenzhen, China). Serum samples were prepared for the determination of cytokines including TNF-*α*, IL-4, and TGF-*β*1. Furthermore, the right lung tissues were fixed in 10% formaldehyde. The tissues were stained with H&E and imaged by a microscope (Leica, Wetzlar, Germany).

### 2.9. Data Analysis

All experiments were repeated at least three times. The analysis of statistical significance was performed utilizing the GraphPad Prism 6.0 Software (GraphPad Software, San Diego, CA). One-way ANOVA with Dunnett's multiple comparison test was used to perform group comparisons. *P* value lower than 0.05 (*P* < 0.05) was used for significant differences.

### 2.10. Reagents

B4 (purity > 99%) was purchased from Jiangxi Bencao Tiangong Technology Co., Ltd. (Nanchang, China, LOT NO. 2018042205). Bacterial lipopolysaccharides (O111:B4), Griess reagent, MTT, JC-1 staining reagent, and DMSO were purchased from Sigma-Aldrich (St. Louis, MO, USA). DMEM, RPMI 1640, penicillin, and streptomycin, FBS, HA-tag magnetic IP/Co-IP kit, TurboFect Transfection Reagent (R0531), and protein A/G magnetic bead kit were obtained from Life Technologies/Gibco Laboratories (Grand Island, NY, USA). ELISA kits for IL-1*β*, TNF-*α*, and IL-6 were purchased from Neobioscience (Shenzhen, China). Antibodies against iNOS (#D6B6), COX-2 (#4842), NLRP3 (#13158), TLR4 (#14358), and GAPDH (#5174) were purchased from Cell Signaling Technology (Beverly, MA, USA); caspase-1 (ab207802) was obtained from Abcam (Cambridge, UK).

## 3. Results

### 3.1. B4 Decreased Proinflammatory Responses in Macrophages

**Figure 1:**
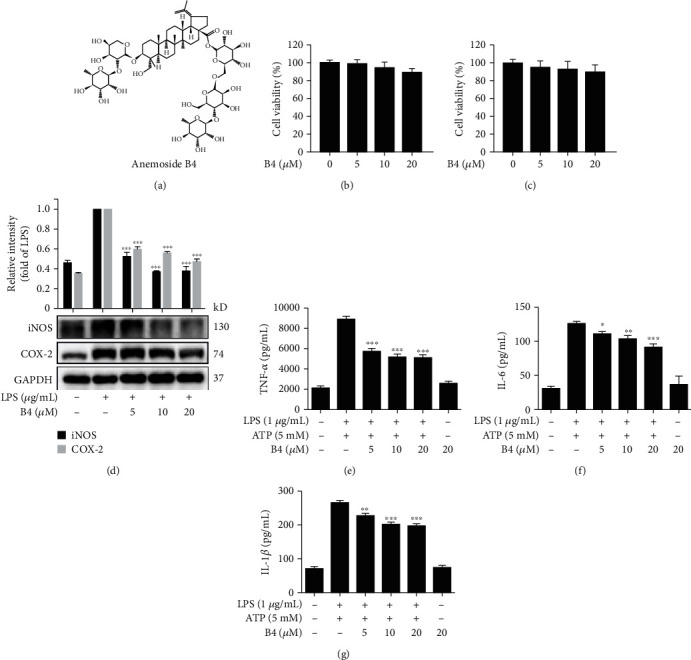
B4 decreased proinflammatory response in macrophages. (a) Chemical structure of B4. (b, c) J774A.1 and THP-1 cells were treated with B4 (5, 10, and 20 *μ*M) for 24 h, and the cell viability was detected by MTT assay. (d) THP-1 cells were pretreated with B4 (5, 10, and 20 *μ*M) for 4 h and subsequently cocultured with LPS (1 *μ*g/mL) for an additional 18 h. Western blotting assay was used to detect the expression of iNOS and COX-2. (e–g) THP-1 cells treated with B4 for 4 h and cocultured with LPS (1 *μ*g/mL) for 5 h, then stimulated with ATP (5 mM) for 30 min. The medium was collected, and the levels of TNF-*α*, IL-6, and IL-1*β* were detected by ELISA kit. All the experiments were performed three times independently. ^∗^*P* < 0.05, ^∗∗^*P* < 0.01, and ^∗∗∗^*P* < 0.001 compared with the model group.

As shown in Figure 1(a), the chemical structure of B4 showed that B4 is affiliated to pentacyclic triterpenoid saponins. Using LPS plus ATP-stimulated J774A.1 and THP-1 cell model, we investigated B4's anti-inflammatory activity and mechanisms *in vitro*. The cytotoxicity of B4 in J774A.1 and THP-1 cells was initially determined by MTT assay. The results indicated that B4 displayed no significant cytotoxicity (Figures 1(b) and 1(c)). As shown in Figure 1(d), the inflammatory messengers like iNOS and COX-2 significantly increased in the model groups. B4 decreased LPS-induced expression of iNOS and COX-2. The proinflammatory cytokines like TNF-*α*, IL-6, and IL-1*β* were released in LPS plus ATP-stimulated THP-1 cells. Using ELISA kits, we found that B4 significantly decreased TNF-*α*, IL-6, and IL-1*β* levels (Figures 1(e)–1(g)). These results illustrated that B4 inhibited inflammatory response in macrophages.

### 3.2. B4 Suppressed NLRP3 Inflammasome Activation

**Figure 2:**
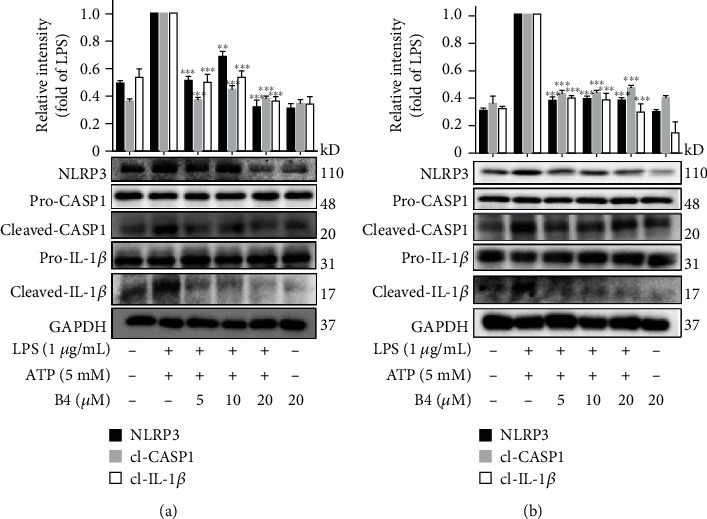
B4 suppressed NLRP3 inflammasome activation. (a, b) J774A.1 cells and THP-1 cells were pretreated with B4 for 4 h, then cocultured with LPS (1 *μ*g/mL) for another 5 h, and stimulated with ATP (5 mM) for 30 min. The expression levels of NLRP3, caspase-1, and IL-1*β* were determined by Western blotting. All the experiments were performed three times independently. ^∗∗^*P* < 0.01 and ^∗∗∗^*P* < 0.001 versus LPS+ATP group.

There are two classical signaling pathways of NLRP3 inflammasome activation. The first signaling pathway activates TLR4 and promotes the transcription of NF-*κ*B, which increased the expression of NLRP3 [39]. The second signaling pathway promotes the formation of the NLRP3/ASC/procaspase-1 complex. The activated caspase-1 then promotes the maturation of pro-IL-1*β* to release the mature IL-1*β* [40]. In this study, we used LPS plus ATP to activate the NLRP3 inflammasome in macrophage cells, and B4 suppressed the proteins' expression of NLRP3 and cleaved caspase-1 and IL-1*β* in LPS plus ATP-stimulated J774A.1 cells and THP-1 cells (Figures 2(a) and 2(b)). These data indicated that B4 suppressed the activation of the NLRP3 inflammasome.

### 3.3. B4 Suppressed the Dysfunction of MMP in LPS Plus ATP-Stimulated Macrophage Cells

**Figure 3:**
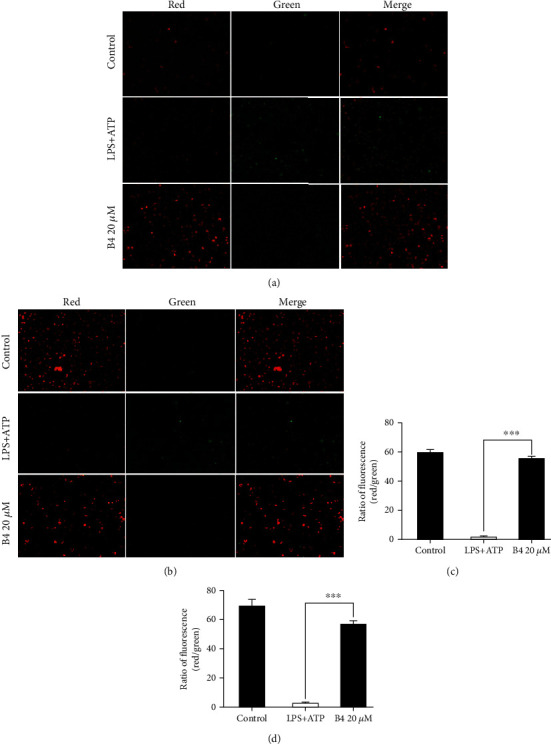
B4 suppressed the dysfunction of MMP in LPS plus ATP-stimulated macrophage cells. (a, b) J774A.1 cells and THP-1 cells were pretreated with B4 for 4 h, then cocultured with LPS (1 *μ*g/mL) for another 5 h, and stimulated with ATP (5 mM) for 30 min. The MMP levels were detected with JC-1 (10 *μ*g/mL) for 30 min in an incubator in the dark. The images were captured by fluorescence microscopy. (c, d) The ratio of red fluorescence compared with green fluorescence in (a) and (b) was analyzed statistically by ImageJ; ^∗∗∗^*P* < 0.001 compared to the LPS+ATP group.

During the development of several inflammatory diseases, mitochondrial membrane potential depolarization plays a pivotal role in NLRP3 inflammasome [41]. LPS plus ATP-induced NLRP3 activation in J774A.1 cells and THP-1 cells leads to the dysfunction of MMP. JC-1 staining was used to detect the MMP. When the cells' MMP is normal, the fluorescence is red, while when the MMP is destroyed, the fluorescence changes to green [42]. Therefore, the changes in the fluorescence could reflect if the MMP is normal or not. Pretreatment with B4 completely reversed the fluorescence of JC-1 from green to yield red in LPS plus ATP-stimulated J774A.1 cells and THP-1 cells (Figures 3(a)–3(d)), suggesting that B4 exhibited a protective effect on MMP loss.

### 3.4. B4 Blocked the Formation of TLR4 Dimerization

**Figure 4:**
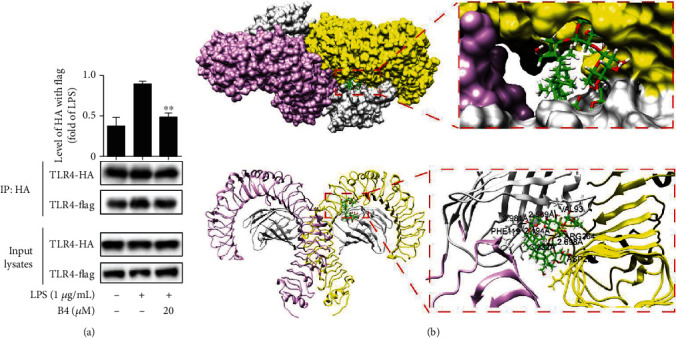
B4 blocked TLR4 dimerization. (a) The two plasmids TLR4-HA and TLR4-Flag were cotransfected into HEK293T cells for 24 h, then pretreated with B4 for 1 h, and cultured with LPS for 12 h. Cells were harvested, and Western blotting was used to analyze the formation of TLR4 dimerization. The experiments were performed three times independently. (b) The molecular docking data of B4 on TLR4-MD-2 dimer were analyzed by using LeDock software. The molecular docking data of B4 with TLR4-MD-2-LPS complex (PDB code: 3FXI) was performed in LeDock (http://www.lephar.com). TLR4-MD-2-LPS complex structures were obtained from the RCSB Protein Data Bank (PDB code: 3FXI). ^∗∗^*P* < 0.01 compared to the LPS group.

TLR4 is a receptor of LPS, which locates on the cell membrane and regulates the inflammatory process. After LPS stimulation, TLR4 will form a dimer, then boost the secretion of proinflammatory cytokines, which has a positive effect on the activation of the NLRP3 inflammasome [43]. In this study, we use TLR4-HA and TLR4-Flag plasmids and the Co-IP assay to detect the TLR4 dimerization after being treated with B4. As shown in Figure 4(a), B4 sharply lessened TLR4-Flag's precipitation, which was pulled down by TLR4-HA, suggesting that B4 blocked TLR4 dimerization. The molecular docking data of B4 on TLR4-MD-2 dimer were analyzed by using LeDock software. The TLR4-MD-2-LPS complex (PDB code: 3FXI) was performed in LeDock (http://www.lephar.com). TLR4-MD-2-LPS complex structures were obtained from the RCSB Protein Data Bank (PDB code: 3FXI). The B4 structure was processed by ChemoDraw 3D, then LeDock was used for the structures of TLR4-MD-2 dimer and B4 to determine the combination of B4 and TLR4-MD-2 dimer, and Pymol was used to analyze the combined energy and site. The molecular docking results identified that B4 occupied the TLR4 dimerizations' pocket (Figure 4(b)) and acted upon VAL93, PHE119, ARG264, and ASP294. Collectively, B4 effectively disturbed TLR4 dimerization.

### 3.5. B4 Rescued LPS-Induced Septic Death and Prevented LPS-Induced ALI in Mice

**Figure 5:**
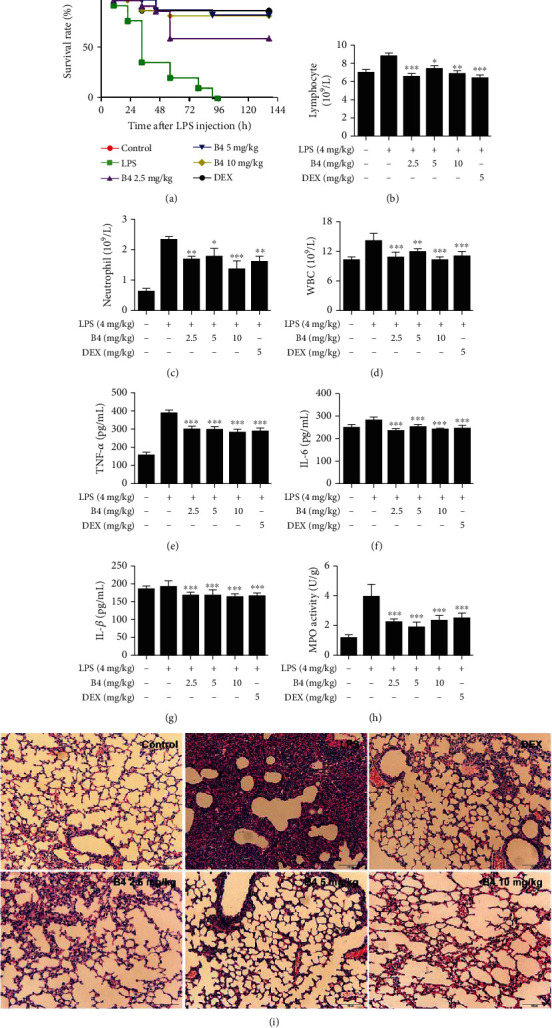
B4 rescued LPS-induced septic death and prevented LPS-induced ALI in mice. (a) Mice of experiment groups were treated with LPS (15 mg/kg, i.t.), and the B4-treated groups were injected with B4 (2.5, 5, and 10 mg/kg, i.v.) at 0, 3, 24, 48, and72 h after LPS administration. DEX group mice were injected with DEX (5 mg/kg, i.p.) after LPS treatment. The survival rate was recorded for the next 144 h (*n* = 20). (b–d) The mice were treated with LPS (4 mg/kg, i.t.). After LPS injection, mice were treated with B4 at 0, 3, 24, 48, and 72 h. After 72 h, blood samples were collected. Blood lymphocytes, neutrophils, and WBC were examined by the blood analyzer (*n* = 20). (e–g) The inflammatory cytokines TNF-*α*, IL-6, and IL-1*β* in serum were detected by ELISA (*n* = 10). (h) The MPO level in lung tissues was detected by MPO kit (*n* = 10). (i) H&E staining of the lung tissue (H&E, original magnification, 200x). DEX (5 mg/kg, i.p.) was used as a positive control (*n* = 10). ^∗^*P* < 0.05, ^∗∗^*P* < 0.01, and ^∗∗∗^*P* < 0.001, compared to LPS-alone group.

LPS-induced sepsis is a commonly used animal model for simulating clinical disease caused by injury or infection [44, 45]. In our study, we explored the effect of B4 on the septic model. The septic model was successfully established by noninvasive intratracheal instillation of LPS (15 mg/kg) into the lung of mice. All mice died within 96 h in the LPS-induced sepsis group. However, B4 (5, 10 mg/kg) obviously increased the mice's survival rate (80%) in 144 h (Figure 5(a)), of which the effect was similar to that of the positive drug, DEX. Even the lower dose of B4 (2.5 mg/kg) exhibited a significant protective effect on LPS-induced septic death.

ALI is a clinical disease with no effective drugs for its treatment. LPS-induced ALI in mice has been identified as the most widespread application of the ALI animal model [46]. LPS increases the release of inflammatory cytokines, such as TNF-*α*, IL-6, and IL-1*β*, which aggravates the development of ALI inflammation [47]. In our study, the ALI model was successfully established by noninvasive intratracheal instillation of LPS (4 mg/kg) into the lung of mice. B4 significantly recovered mice's lung function and decreased LYMPH (Figure 5(b)), NEUT (Figure 5(c)), WBC (Figure 5(d)), TNF-*α* (Figure 5(e)), IL-6 (Figure 5(f)), IL-1*β* (Figure 5(g)), and myeloperoxidase (MPO) (Figure 5(h)) in LPS-induced ALI mouse models. The H&E staining displayed that the LPS-challenged mice caused obvious interstitial exudation of alveoli, the destruction of normal structure of alveoli, and the infiltration of a large number of inflammatory cells. These lesions of lung tissues were improved by B4, as well as the DEX-positive control drug (Figure 5(i)). Collectively, B4 rescued LPS-induced ALI in the mouse model.

### 3.6. B4 Rescued BLM-Stimulated ALI in Mice

**Figure 6:**
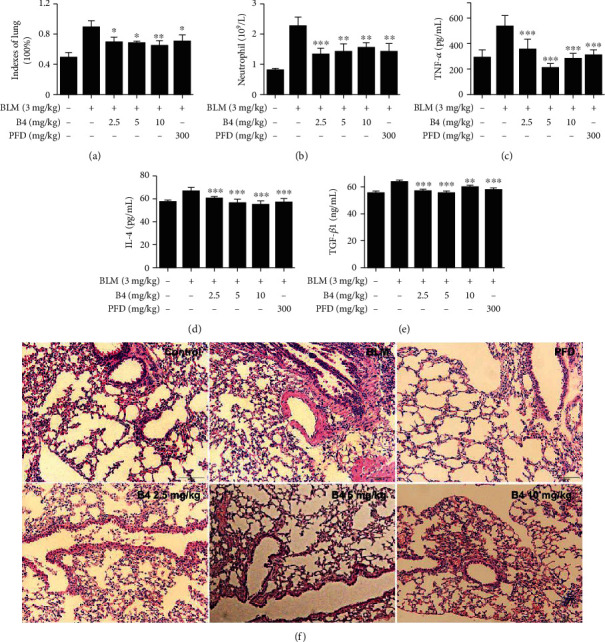
B4 rescued BLM-stimulated ALI in mice. (a) Mice were treated with BLM (3 mg/kg, i.t.). After BLM injection, mice were injected with B4 at 0, 3, 24, and 48 h. PFD group mice were administrated with PFD (300 mg/kg, i.g.) at 0, 3, 24, and 48 h after BLM treatment. Lung tissues were weighed, and lung index was calculated (*n* = 20). (b) Blood samples were collected. Blood neutrophils were examined by the blood analyzer, (*n* = 20). (c–e) The serum inflammatory cytokines TNF-*α*, IL-4, and TGF-*β*1 were detected by ELISA (*n* = 10). (f) H&E staining of the lung tissue (H&E, original magnification, 200x). PFD (300 mg/kg, i.p.) was the positive control (*n* = 10). ^∗^*P* < 0.05, ^∗∗^*P* < 0.01, and ^∗∗∗^*P* < 0.001, compared to the BLM-alone group.

BLM has been identified as another factor that leads to ALI [48]. BLM promotes the section of TNF-*α*, IL-4, and TGF-*β*1, which contributes to the process of ALI and destruction of lung function [49]. In this study, B4 significantly rescued the mouse lung index (Figure 6(a)) and decreased BLM-induced NEUT (Figure 6(b)), TNF-*α* (Figure 6(c)), IL-4 (Figure 6(d)), and TGF-*β*1 (Figure 6(e)) in mouse models. H&E staining displayed BLM-challenged mice with obvious interstitial exudation of alveoli, the destruction of the normal structure of alveoli, and the infiltration of a large number of inflammatory cells. These lesions of lung tissues were improved by B4, as well as by a PFD-positive control drug (Figure 6(f)). Collectively, B4 has a significant protective effect on the BLM-induced mouse ALI model.

## 4. Discussion

ALI is an acute inflammatory reaction, which leads to microvascular injury to increase pulmonary vascular endothelium and alveolar epithelium's permeability and causes ARDS at a later stage [50]. During ALI, the secretion of TNF-*α*, IL-6, and IL-1*β* in macrophages such as J774A.1 and THP-1 cells affects the expression of iNOS and COX-2 [51]. The iNOS can promote the production of NO, which eliminates the pathogen but, at the same time, aggravates the ALI [52]. For LPS-stimulated macrophages, iNOS and COX-2 will be overexpressed to increase the secretion of proinflammatory cytokines like TNF-*α*, IL-6, and IL-1*β* during the inflammatory process [53]. B4, a compound isolated from *Pulsatilla chinensis* (Bunge) Regel, exhibits a deluge of bioactivities [34, 54, 55]. As it stands now, no more than 30 published papers involved in B4 are found, which mainly focus on its quality control and chemical analysis. Quite a few studies uncover B4's bioactivity. Gong et al. illustrated that B4 exhibited a protective effect on rat kidney injury *via* suppressing inflammation [56]. In addition, B4 showed nephrotoxicity of cisplatin without cytotoxic effect on HEK293T cells [35]. However, Xue et al. found that B4 exhibited significant cytotoxicity in SMMC7721 cells [57]. It is of interest to further investigate the cytotoxicity of B4. In our study, we found that B4 showed no cytotoxicity in macrophages and HEK293T cells. A previous study demonstrated that B4 exhibited anti-inflammatory activity *in vivo* [36]. However, the detailed mechanism of anti-inflammatory activity and the therapeutic effect of B4 on ALI have not been reported. In this study, we initially found that B4 significantly suppressed iNOS and COX-2 expression in LPS-induced THP-1 cells, NLRP3 inflammasome activation in J774A.1 cells and THP-1 cells, and TLR4 dimerization.

ALI always happens when NLRP3 inflammasome is overactivated, which promotes the proinflammatory cytokines' release like TNF-*α*, IL-6, IL-1*β*, MPO, and the loss of MMP [58]. Upon the activation of the NLRP3 inflammasome, the expression of cleaved caspase-1 and mature IL-1*β* is overexpressed. The signals of LPS plus ATP-stimulated NLRP3 inflammasome disrupt the balance of K^+^, Ca^2+^, and MMP, which promote the generation of reactive oxygen species (ROS) [59–61]. Therefore, inhibiting the NLRP3 inflammasome activation may provide an alternative strategy for treatment with ALI. In this study, our results showed that B4 inhibited the activation of NLRP3 inflammasome through decreasing the expression of NLRP3, caspase-1, and IL-1*β* in LPS plus ATP-induced macrophages.

TLR4 is the first pattern-recognition receptors (PRRs) [62]. When TLR4 is stimulated by LPS, a common “pattern” structure heterodimer TLR4-MD-2 forms a horse-shoe-like-shape dimer, which initially activates proinflammatory signaling pathways. Activated TLR4 will regulate NLRP3 inflammasome during ALI. Thus, inhibiting the TLR4 dimer formation is a potential strategy to treat ALI. In this study, we found that B4 significantly suppressed the TLR4 dimerization *via* binding to the active sites of TLR4-MD-2-LPS.

To further confirm B4's therapeutic effects on ALI, we used three animal models, including LPS-induced septic mouse, LPS-induced ALI, and BLM-induced ALI models. All animal models were successfully established by noninvasive intratracheal instillation of LPS or BLM into the lung of mice. In our study, B4 increased mice's survival rate. The therapeutic effects of B4 at a higher dosage of 5 and 10 mg/kg were similar to those of DEX. Even B4 at a lower dosage of 2.5 mg/kg exhibited a significant protective effect on LPS-induced septic death, suggesting that B4 has the same effect of DEX in the absence of DEX's side effects, which is a potential lead compound for the treatment of ALI. In addition, B4 prevented ALI by recovering the lung functions and inhibiting the secretion of proinflammatory factors. B4 suppressed the WBC, NEUT, and LYMPH in the blood, decreased IL-1*β*, IL-6, and TNF-*α* levels in serum, and inhibited the activation of MPO in lung tissues. Specifically, H&E staining results indicated that B4 significantly prevented ALI. Collectively, B4 showed a protective effect on LPS-induced ALI. In addition to the LPS-induced ALI model, we also used the BLM-induced ALI mouse model to investigate B4's therapeutic effect on ALI. Similar to the results of LPS-induced ALI, B4 exhibited a significant protective effect on BLM-induced ALI. BLM upregulated the generation of TNF-*α*, IL-4, and TGF-*β*1 in serum and the level of NEUT in mice, which were reversed by B4. H&E staining analysis showed that B4 successfully ameliorated BLM-induced ALI. Taken together, B4 prevented LPS- and BLM-induced ALI.

## 5. Conclusions

In summary, our study indicated that B4 protected ALI through attenuating inflammation *via* blocking NLRP3 inflammasome activation and TLR4 dimerization.

## Data Availability

The Anemoside B4 protects acute lung injury by attenuating inflammation through blocking NLRP3 inflammasome activation, and the TLR4 dimerization data used to support the findings of this study are included within the article.

## Abbreviations

LPS: Lipopolysaccharides
NLRP3: Nucleotide-binding domain, leucine-rich-repeat-containing protein 3 (NOD-like receptor 3)
TLR4: Toll-like receptor 4
TNF-*α*: Tumor necrosis factor-*α*
COX-2: Cyclooxygenase-2
iNOS: Inducible nitric oxide synthase
IL-1*β*: Interleukin-1*β*
IL-6: Interleukin-6
IL-4: Interleukin-4
TGF-*β*1: Transforming growth factor beta 1
ALI: Acute lung injury
H&E: Hematoxylin and eosin
DEX: Dexamethasone
BLM: Bleomycin
PFD: Pirfenidone.
